# Virulence-inhibitory activity of the degradation product 3-hydroxybutyrate explains the protective effect of poly-β-hydroxybutyrate against the major aquaculture pathogen *Vibrio campbellii*

**DOI:** 10.1038/s41598-018-25385-w

**Published:** 2018-05-08

**Authors:** Tom Defoirdt, Nguyen Thi Mai Anh, Peter De Schryver

**Affiliations:** 1 Center for Microbial Ecology and Technology, Ghent University, Coupure Links 653, Ghent, Belgium; 2 Laboratory of Aquaculture & Artemia Reference Center, Gent University, Coupure Links 653, Ghent, Belgium; 3 Research Institue in Aquaculture no 2, Ho Chi Minh City, Vietnam; 4 INVE Technologies NV, Hoogveld 93, Dendermonde, Belgium

## Abstract

The bacterial storage compound poly-β-hydroxybutyrate, a polymer of the short-chain fatty acid 3-hydroxybutyrate, has been reported to protect various aquatic animals from bacterial disease. In order to obtain a better mechanistic insight, we aimed to (1) investigate whether 3-hydroxybutyrate is released from poly-β-hydroxybutyrate within sterile brine shrimp larvae, (2) determine the impact of 3-hydroxybutyrate on the virulence of *Vibrio campbellii* to brine shrimp larvae and on its cell density in the shrimp, and (3) determine the impact of this compound on virulence factor production in the pathogen. We detected 3-hydroxybutyrate in poly-β-hydroxybutyrate-fed brine shrimp, resulting in 24 mM 3-hydroxybutyrate in the intestinal tract of shrimp reared in the presence of 1000 mg l^−1^ poly-β-hydroxybutyrate. We further demonstrate that this concentration of 3-hydroxybutyrate does not affect the growth of *V*. *campbellii*, whereas it decreases the production of different virulence factors, including hemolysin, phospholipase and protease activities, and swimming motility. We hypothesize that by affecting all these virulence factors at once, 3-hydroxybutyrate (and thus also poly-β-hydroxybutyrate) can exert a significant impact on the virulence of *V*. *campbellii*. This hypothesis was confirmed in a challenge test showing that 3-hydroxybutyrate protected gnotobiotic brine shrimp from pathogenic *V*. *campbellii*, without affecting the number of host-associated vibrios.

## Introduction

Aquaculture is playing an increasingly important role with respect to food security, and the sector is predicted to dominate aquatic food supply within a few years^1^. However, the further sustainable expansion of the aquaculture sector is currently hampered by a number of factors, amongst which diseases are playing a prominent role, especially in the early life stages of the animals^2^. Vibrios belonging to the *Harveyi* clade (such as *Vibrio campbellii*) are amongst the major pathogens of marine organisms^3^. These bacteria cause huge losses in the aquaculture industry worldwide, with acute hepatopancreatic necrosis disease (AHPND) as a notable recent example^4,5^. Global losses in the shrimp farming industry due to this disease have been estimated to be over US$ 1 billion per year during the last eight years^6^. The wide and frequent use of antibiotics has resulted in the development and spread of (multiple) antibiotic resistance in aquaculture pathogens, rendering antibiotics ineffective in treating bacterial diseases^7^. Moreover, public opinion currently distrusts the use of antibiotics in food production^8^, and therefore, alternatives to antibiotics are needed in order to enable the aquaculture sector to further expand in a sustainable way. One of the strategies that is currently receiving significant attention in the field, is antivirulence therapy – the inhibition of virulence, rather than growth, of bacterial pathogens^9^.

Short-chain fatty acids are able to inhibit the growth of various bacteria, and as a consequence, there is interest in the use of these compounds as biocontrol agents in animal production^10^. These compounds are also able to protect aquatic animals from vibriosis^11^. However, because of dilution in the aquatic environment, the effective doses need to be high, rendering short-chain fatty acids economically less attractive for disease control in aquaculture^12^. In order to overcome this limitation, we have used the insoluble bacterial storage compound poly-β-hydroxybutyrate, a polymer of the short-chain fatty acid 3-hydroxybutyrate, as a source of short-chain fatty acids^13^. Abiotic decomposition of poly-β-hydroxybutyrate is a relatively slow process under mild conditions and the degradation rate is affected by factors such as pH and temperature^12^. Poly-β-hydroxybutyrate can be degraded in the gastrointestinal tract of animals. Indeed, Freier *et al*. reported that digestive enzymes increased the decomposition of poly-β-hydroxybutyrate threefold in phosphate buffer^14^. Forni *et al*. reported that untreated poly(β-hydroxybutyrate-co-β-hydroxyvalerate) copolymer was poorly digested in pigs^15^. However, poly-β-hydroxybutyrate is most efficiently degraded by poly-β-hydroxybutyrate depolymerase enzymes produced by various micro-organisms, and this enzyme activity also results in an improved protective effect of poly-β-hydroxybutyrate against vibriosis in aquatic animals^16^.

We have previously demonstrated that the addition of poly-β-hydroxybutyrate to the rearing water offers protection against *V*. *campbellii* in a model system with brine shrimp larvae^13^. Later research revealed that poly-β-hydroxybutyrate improved the survival of various aquaculture species, including finfish, crustaceans as well as mollusks^17–22^. Poly-β-hydroxybutyrate is water-insoluble, and therefore, we hypothesized that in order to exert a beneficial effect, the polymer must be degraded into water-soluble products (e.g. 3-hydroxybutyrate monomers) in the intestinal tract, and that these water-soluble products are responsible for the beneficial effect^12^. This hypothesis was based on indirect observations because thus far, the release of 3-hydroxybutyrate in poly-β-hydroxybutyrate-fed animals has not been demonstrated.

Short-chain fatty acids, including 3-hydroxybutyrate, are capable of inhibiting the growth of vibrios belonging to the *Harveyi* clade in a pH-dependent manner, with effective concentrations within the range of 10–100 mM^11,13^. The pH-dependence of the effect is due to the fact that the short-chain fatty acids can only pass through the cell membranes of bacteria in their undissociated form^10^, which is more prevalent at lower pH (according to the Henderson-Hasselbach equation). Once inside the cytoplasm, the short-chain fatty acids dissociate, thereby increasing the intracellular concentration of protons^23^. This is thought to be the reason for the growth-inhibitory effect of short-chain fatty acids because the cells need to spend energy in order to maintain the intracellular pH at the optimal level and thus cannot use this energy for other metabolic processes^10^. Because short-chain fatty acids are thought to interfere with the energy resources of bacteria, a stronger growth-inhibitory effect is expected in nutrient-poor environments than in nutrient-rich environments (where energy resources are more abundant). Remarkably, we previously found that short-chain fatty acids (including 3-hydroxybutyrate) protect gnotobiotic brine shrimp from *V*. *campbellii* at concentrations that are below the growth-inhibitory concentration^11,13^. One possible explanation for this observation might be that sub-growth-inhibitory concentrations of short-chain fatty acids decrease virulence gene expression in the pathogen, and in this way prevent it from infecting the host^12^. Indeed, short-chain fatty acids have been shown before to decrease virulence factor production in enteric pathogens such as *Salmonella enterica* and Shiga toxin-producing *Escherichia coli*^24–26^. However, the impact of 3-hydroxybutyrate on virulence factor production in vibrios is currently unknown.

In order to obtain a better insight into the protective mechanism of poly-β-hydroxybutyrate, in this study, we aimed at investigating the release of 3-hydroxybutyrate from poly-β-hydroxybutyrate in sterile brine shrimp larvae, to determine the impact of subinhibitory concentrations of 3-hydroxybutyrate that are representative of those released from poly-β-hydroxybutyrate in the host, on virulence factor production by *V*. *campbellii*, and to determine the impact of 3-hydroxybutyrate on the virulence of the pathogen to brine shrimp and on its cell density in the brine shrimp.

## Results

### Determination of 3-hydroxybutyrate levels in poly-β-hydroxybutyrate-fed brine shrimp larvae

We previously hypothesised that the beneficial activity of poly-β-hydroxybutyrate is mediated by the release of water-soluble 3-hydroxybutyrate monomers^12^. In order to investigate this, we set-up sterile brine shrimp cultures with different concentrations of poly-β-hydroxybutyrate particles added to the rearing water, and then determined the concentration of 3-hydroxybutyrate in brine shrimp larvae harvested after 24 h. The concentration of 3-hydroxybutyrate in the brine shrimp samples increased with increasing doses of poly-β-hydroxybutyrate in the rearing water (Table 1). The 3-hydroxybutyrate concentrations in samples from the 100 and 1000 mg l^−1^ poly-β-hydroxybutyrate treatments were significantly different from those in samples taken from brine shrimp cultures that had not received poly-β-hydroxybutyrate. Assuming that all 3-hydroxybutyrate originated from the intestinal tracts of the sampled larvae, we further calculated the concentration of 3-hydroxybutyrate in the brine shrimp gut, and found that 3-hydroxybutyrate concentrations increased to 24 mM in the presence of 1000 mg l^−1^ poly-β-hydroxybutyrate (Table 1).

**Table 1:** Concentration of 3-hydroxybutyrate (3HB) in sterile brine shrimp (*Artemia franciscana*) samples that received different concentrations of poly-3-hydroxybutyrate (PHB) in the rearing water (average ± standard deviation of 3 replicates).

[PHB] added (mg l^−1^)	[3HB] in samples (µg ml^−1^)	Estimated [3HB] in the gut (mM)
0	12 ± 5	9 ± 4
10	16 ± 1	11 ± 1
100	24 ± 0*	17 ± 0
1000	35 ± 4*	24 ± 3

The concentrations of 3HB in the brine shrimp gut were calculated as described in Materials and Methods. Asterisks indicate significantly higher concentrations than in the 0 mg l^−1^ PHB treatment (*: P < 0.05).

### Impact of 3-hydroxybutyrate on the growth of *Vibrio campbellii*

**Figure 1:**
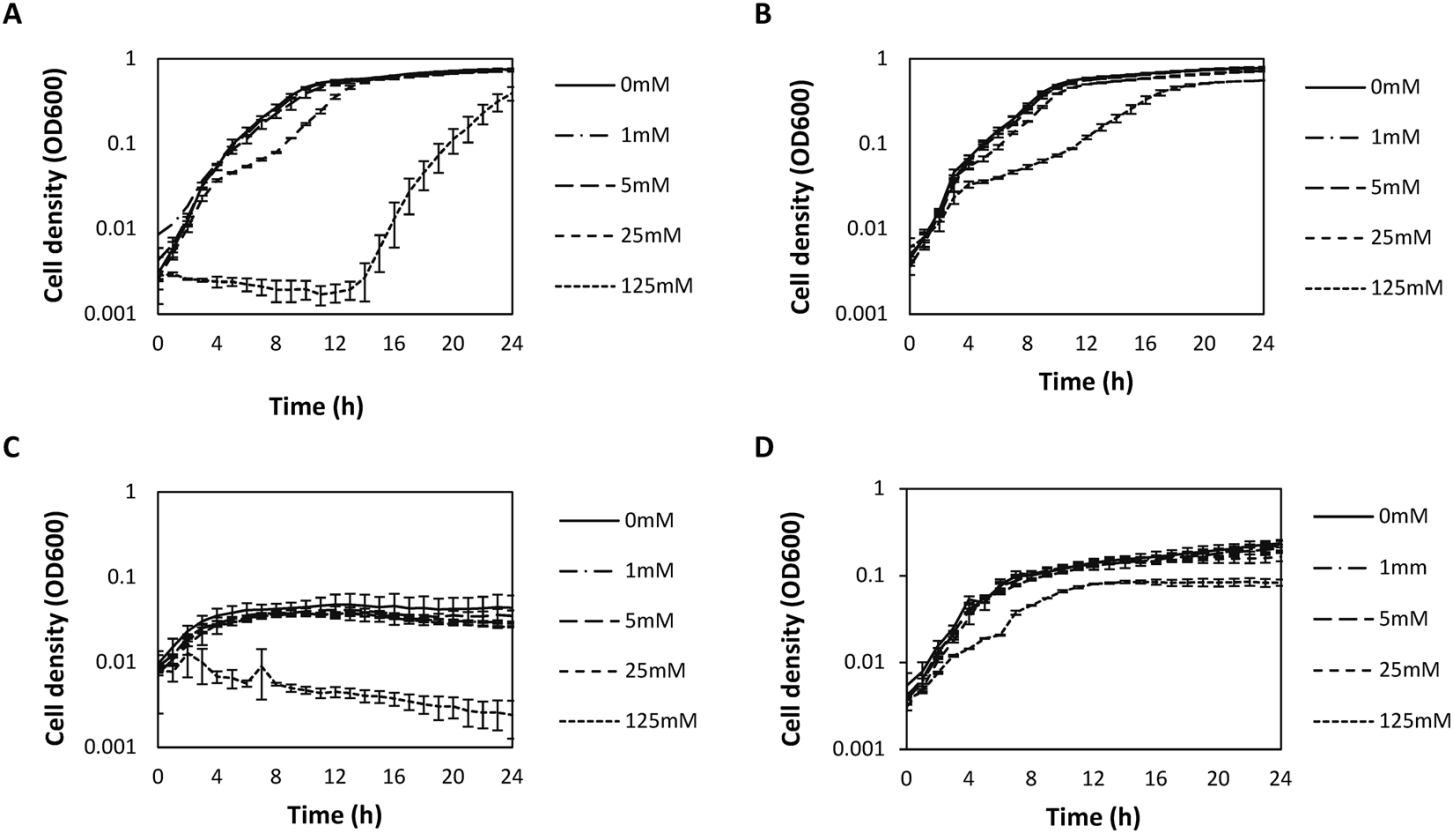
Growth of *Vibrio campbellii* ATCC BAA-1116 in nutrient-rich (panels A and B) and nutrient-poor (panels C and D) media at pH 6 (panels A and C) or pH 7 (panels B and D) in the presence of different concentrations of 3-hydroxybutyrate. The error bars represent the standard deviation of three replicates.

We investigated the impact of 3-hydroxybutyrate on the growth of *V*. *campbellii* in both nutrient-rich and nutrient-poor conditions, and at pH 6 or 7. At pH 6, the growth of *V*. *campbellii* was inhibited at the highest concentration of 3-hydroxybutyrate (125 mM), with a significantly longer lag phase in nutrient-rich conditions and a complete inhibition of growth in nutrient-poor conditions (Fig. 1A and C). At pH7, on the other hand, only a relatively small effect was observed at 125 mM 3-hydroxybutyrate, both in nutrient-rich and nutrient-poor conditions (Fig. 1B and D).

### Impact of 3-hydroxybutyrate on the survival of brine shrimp larvae challenged with *Vibrio campbellii* and on the cell density of shrimp-associated vibrios

**Figure 2:**
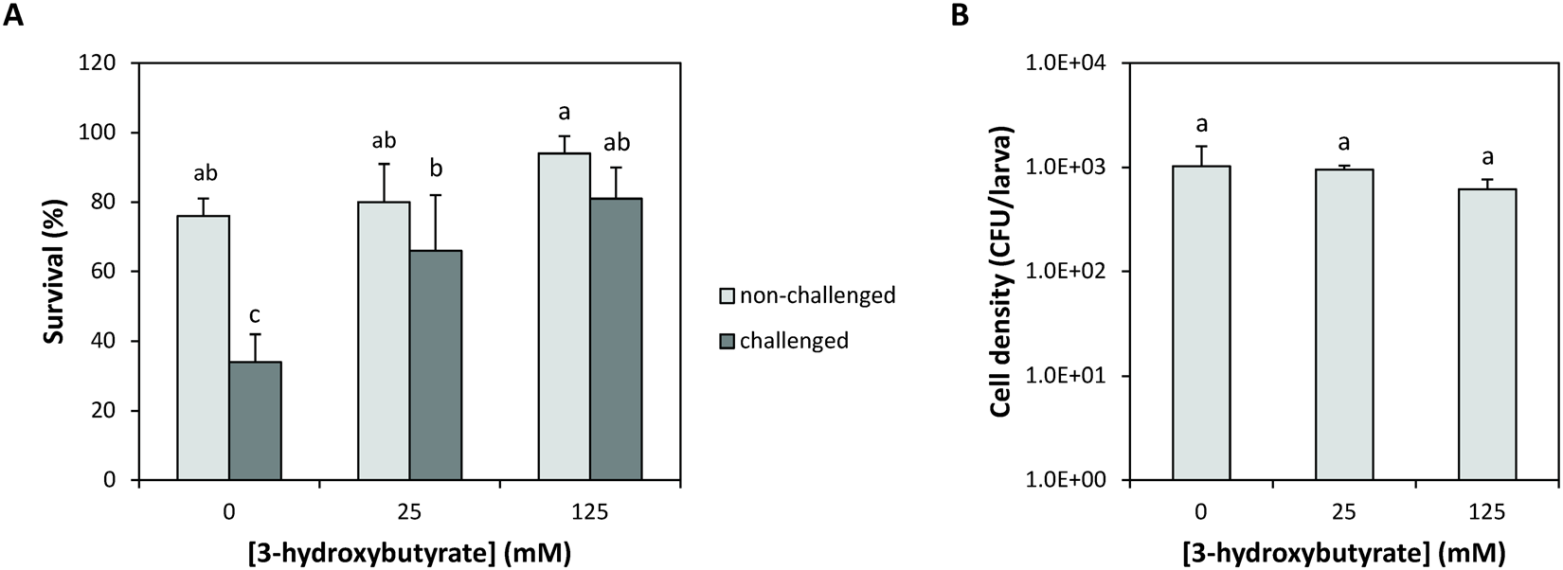
(**A**) Survival of gnotobiotic brine shrimp (*Artemia franciscana*) larvae after 2 days of incubation with or without 3-hydroxybutyrate and with or without *Vibrio campbellii* ATCC BAA-1116 challenge. (**B**) Density of *Vibrio campbellii* ATCC BAA-1116 associated with live brine shrimp larvae at the end of the challenge test. Error bars represent the standard error of four shrimp cultures. Bars with a different superscript letter are significantly different from each other (One way ANOVA with Duncan’s posthoc test, P < 0.01).

In a further experiment, we determined the impact of 25 or 125 mM 3-hydroxybutyrate on the virulence of *V*. *campbellii* in a standardised model system with gnotobiotic brine shrimp larvae. We have previously demonstrated that the addition of 1000 mg l^−1^ poly-β-hydroxybutyrate to the rearing water offers protection against *V*. *campbellii* in this model system^13^. Brine shrimp larvae are particle filter feeders that are unable to accumulate dissolved compounds^27^ and therefore, the concentration of 3-hydroxybutyrate within the brine shrimp larvae is expected to be the same as the concentration in the rearing water. Larvae challenged with *V*. *campbellii* showed a significantly higher survival in the presence of both concentrations of 3-hydroxybutyrate, and the survival of challenged larvae in the presence of 3-hydroxybutyrate was not significantly different from that of unchallenged larvae (Fig. 2A). We also observed that the survival of non-challenged larvae was higher in the presence of 3-hydroxybutyrate, suggesting a direct beneficial effect on the brine shrimp. Finally, we determined the numbers of *V*. *campbellii* that were associated with the brine shrimp larvae and found that there were no significant differences between treatments (Fig. 2B).

### Impact of 3-hydroxybutyrate on virulence factor production by *Vibrio campbellii*

**Figure 3:**
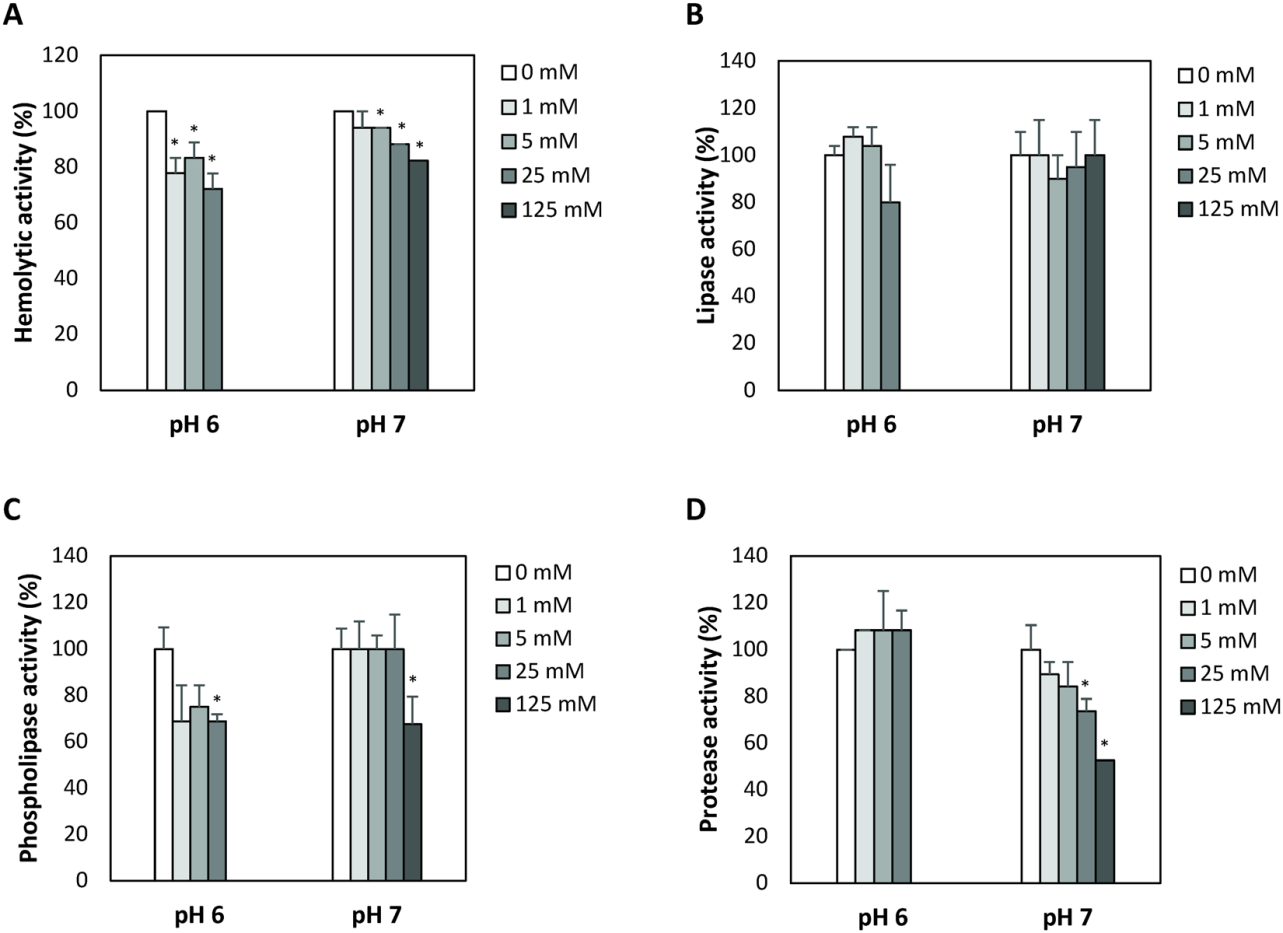
Hemolytic (panel A), lipase (panel B), phospholipase (panel C) and protease (panel D) activities of *Vibrio campbellii* ATCC BAA-1116 in the presence of different concentrations of 3-hydroxybutyrate (0, 1, 5, 25 and 125 mM, respectively), at pH 6 and 7. The activities without 3-hydroxybutyrate were set at 100% and all other treatments were normalized accordingly. Error bars represent the standard deviation of three replicates. Asterisks indicate significant differences with the treatment without 3-hydroxybuturate (P < 0.05). Treatment with 125 mM 3-hydroxybutyrate was not tested at pH 6.

**Figure 4:**
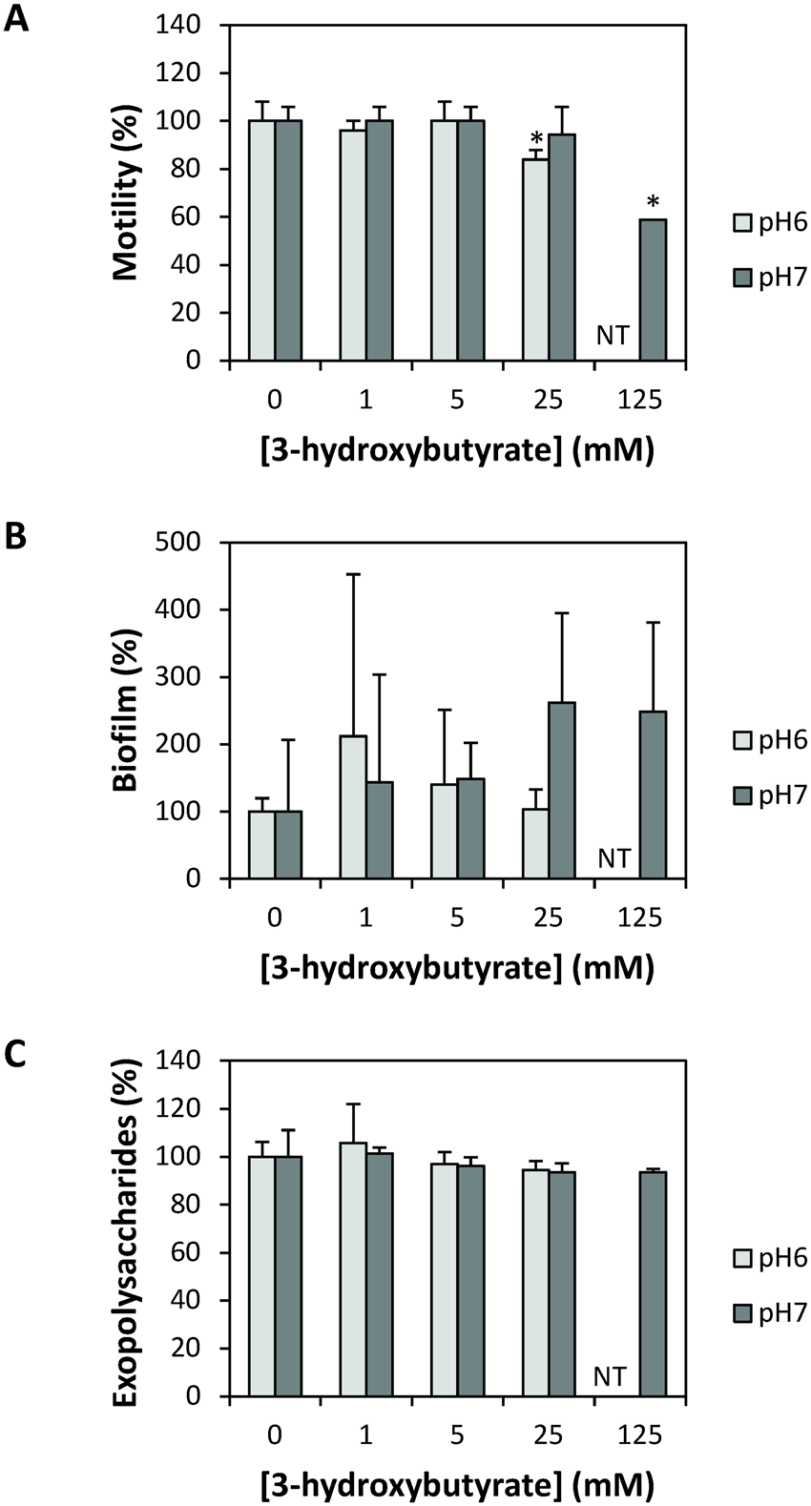
Motility (panel A), biofilm formation (panel B) and exopolysaccharide production (panel C) of *Vibrio campbellii* ATCC BAA-1116 in the presence of different concentrations of 3-hydroxybutyrate, at pH6 or pH7. Error bars represent the standard deviation of three replicates. Bars that are significantly different from the treatment without 3-hydroxybutyrate are marked with an asterisk (P < 0.05). NT: Not Tested.

We determined the impact of non-growth inhibitory concentrations of 3-hydroxybutyrate on the production of hemolytic, lipase, phospholipase and protease activities in *V*. *campbellii*. Hemolytic activity decreased at 1 mM of 3-hydroxybutyrate or more, with a maximal decrease of 28 and 18% at pH 6 and 7, respectively (Fig. 3A). Lipase activity was not affected by 3-hydroxybutyrate (Fig. 3B). Phospholipase activity decreased in the presence of 25 mM 3-hydroxybutyrate at pH6 and in the presence of 125 mM at pH 7 (Fig. 3C). Protease activity was decreased only at pH 7, with a 26% decrease in the presence of 25 mM 3-hydroxybutyrate, and no clearing zone extending beyond the colony diameter in the presence of 125 mM 3-hydroxybutyrate (Fig. 3D). Swimming motility decreased by 16% in the presence of 25 mM 3-hydroxybutyrate at pH 6, and by 41% in the presence of 125 mM 3-hydroxybutyrate at pH 7 (Fig. 4A). Finally, there was no impact of 3-hydroxybutyrate on biofilm formation and exopolysaccharide production at any of the concentrations tested (Fig. 4B and C).

## Discussion

During recent years, research has revealed that the bacterial storage compound poly-β-hydroxybutyrate, a polymer of the short-chain fatty acid 3-hydroxybutyrate, is able to improve the survival of various aquaculture species^13,17–22^. Because poly-β-hydroxybutyrate is water-insoluble, we have hypothesized that in order to exert a beneficial effect, the polymer must be degraded into water-soluble products (e.g. 3-hydroxybutyrate monomers) in the intestinal tract of aquatic animals, and that these water-soluble products are responsible for the beneficial effect^12^. This hypothesis was based on indirect observations including (1) increased survival of sterile starved brine shrimp in the presence of poly-β-hydroxybutyrate, (2) an improved effect of poly-β-hydroxybutyrate in the presence of poly-β-hydroxybutyrate-degrading bacteria, and (3) a decreased intestinal pH in sterile sea bass larvae receiving poly-β-hydroxybutyrate in their diet^13,16,28^. In this study, we demonstrate for the first time that 3-hydroxybutyrate is indeed released in poly-β-hydroxybutyrate-fed animals. Importantly, we used sterile brine shrimp for this experiment, excluding the possibility that the release of 3-hydroxybutyrate is a result of bacterial poly-β-hydroxybutyrate depolymerase activity. Therefore, the release of 3-hydroxybutyrate must be a result of abiotic decomposition and/or enzyme activity in the larvae. Abiotic decomposition in the rearing water is unlikely to explain the (relatively high) levels of 3-hydroxybutyrate we measured in the shrimp samples since the abiotic release of 3-hydroxybutyrate from poly-β-hydroxybutyrate in aqueous solutions is very limited. For instance, Freier *et al*. reported that less than 5% of PHB decomposed abiotically in phosphate buffer at 37 °C after 4 weeks^14^. A similar decomposition rate would have resulted in 3-hydroxybutyrate concentrations in the brine shrimp rearing water below 2, 20 and 200 µg l^−1^ for the 10, 100 and 1000 mg l^−1^ poly-β-hydroxybutyrate treatments, respectively, at the time of taking our samples. Because brine shrimp are particle filter feeders that are not able to accumulate dissolved compounds^27^, the concentration of dissolved compounds (such as 3-hydroxybutyrate) in the shrimp body will be the same as in the water. This means that the 3-hydroxybutyrate concentration resulting from abiotic decomposition in the rearing water would have resulted in concentrations in the shrimp that are lower than 2, 20 and 200 µg l^−1^. This is more than 175 times lower than the concentrations we measured in our samples. Previous research has also shown that poly-β-hydroxybutyrate decomposes in the digestive tract of animals^15^ and that digestive enzymes increase the abiotic decomposition of the compound^14^.

We calculated that the 3-hydroxybutyrate concentration in the gut of brine shrimp larvae reared in the presence of 1000 mg l^−1^ poly-β-hydroxybutyrate, increased to 24 mM. This concentration is within the range of concentrations of short-chain fatty acids (including 3-hydroxybutyrate) that have been shown to increase the survival of brine shrimp larvae challenged with *V*. *campbellii* LMG 21363^11,13^, and this was confirmed here for *V*. *campbellii* ATCC BAA-1116. The effect we observed here for strain ATCC BAA-1116 (complete protection) was more pronounced than what we found previously for strain LMG 21363^13^, which reflects the fact that strain LMG 21363 is more virulent than strain ATCC BAA-1116^29^.

In order to obtain a further mechanistic insight into the protective effect of the poly-β-hydroxybutyrate degradation product 3-hydroxybutyrate, we subsequently determined the impact of the compound on the growth of *V*. *campbellii*, both in nutrient-rich and nutrient-poor conditions, the latter of which might be more relevant to the conditions experienced in the rearing water and in a host. We expected that the impact would be stronger in nutrient-poor conditions because short-chain fatty acids are thought to interact with the energy balance in bacteria^10^, and since the cellular energy pool is expected to be lower in nutrient-poor conditions. We found that 3-hydroxybutyrate inhibits the growth of the vibrios in a pH-dependent manner, showing inhibition at 125 mM at pH 6 (but not at pH 7), and this effect was indeed more pronounced in nutrient-poor conditions. The effect we observed here is similar to what has been reported before for other short-chain fatty acids in vibrios^11,13^ and other Gram-negative bacteria, such as *Salmonella* spp^25^.

We previously reported that short-chain fatty acids (including 3-hydroxybutyrate) increased the survival of brine shrimp larvae challenged with *V*. *campbellii* LMG 21363 at concentrations that were below the growth-inhibitory concentration^11,13^, and this was confirmed here for *V*. *campbellii* ATCC BAA-1116. Furthermore, we showed here that 3-hydroxybutyrate had no effect on the number of vibrios associated with the brine shrimp larvae, further indicating that the beneficial effect is not caused by inhibition of the growth of the pathogen. Because of this, we hypothesized that the compound might decrease the ability of the vibrios to attack their host, and in order to investigate this, we tested the impact of 3-hydroxybutyrate on a number of virulence-related phenotypes. We found that 3-hydroxybutyrate could indeed decrease hemolytic, phospholipase and protease activities, and swimming motility in *V*. *campbellii*. Consistent with this, Kiran *et al*. recently reported that poly-β-hydroxybutyrate degradation products generated by poly-β-hydroxybutyrate depolymerase activity of *Vibrio* sp. PUGSK8 inhibited hemolytic activity and motility of the bacterium, without affecting its growth^30^. Although the degradation products were not identified in the latter study, poly-β-hydroxybutyrate depolymerases usually degrade poly-β-hydroxybutyrate into 3-hydroxybutyrate oligomers or monomers^31^. Hence, we hypothesize that the effect observed for poly-β-hydroxybutyrate-treated *Vibrio* sp. PUGSK8 was also caused by 3-hydroxybutyrate. Short-chain fatty acids are considered to affect the energy balance of bacterial cells (leading to growth inhibition at a certain concentration)^10^, and given the fact that the production (and activity) of virulence factors is metabolically costly^32,33^, we hypothesize that subinhibitory concentrations of 3-hydroxybutyrate decrease the cellular energy status to a level at which virulence, but not growth, is decreased, thus exerting a non-specific antivirulence effect.

Several putative virulence factors have been proposed in vibrios belonging to the *Harveyi* clade. These include flagellar motility, which enables the pathogens to colonize the host, exopolysaccharides that enable pathogens to form biofilms and to attach to host tissues and protect them from the host’s defense system, and lytic enzymes that degrade host tissues and enable pathogens to obtain nutrients^34^. Amongst the most well-known lytic enzymes are hemolysins (damaging blood cells), lipases (degrading lipids), phospholipases (degrading phospholipids) and proteases (degrading proteins). We previously reported that there is a positive correlation between hemolysin and protease expression, and virulence towards brine shrimp larvae^35^, and that swimming motility is required for full virulence of *V*. *campbellii*^36^. Furthermore, hemolytic and phospholipase activity have also been implicated in virulence of strains belonging to the *Harveyi* clade^37,38^. Hence, by simultaneously decreasing the production of these virulence factors (even though some of the effects were relatively modest), 3-hydroxybutyrate (and thus also poly-β-hydroxybutyrate) can exert a significant impact on the overall virulence of *V*. *campbellii* in the host. Furthermore, Baruah *et al*. recently reported that poly-β-hydroxybutyrate induces protective innate immune responses in gnotobiotic brine shrimp larvae challenged with *V*. *campbellii*, including an increase in phenoloxidase activity, one of the major innate immune responses of crustaceans^39^. It would be highly interesting to unravel which property of poly-β-hydroxybutyrate is responsible for this effect as it might also be mediated by a degradation product of the polymer (such as 3-hydroxybutyrate). Hence, we think that the strong protective effect of poly-β-hydroxybutyrate against bacterial infections can be explained by both a decreased virulence of the pathogens (caused by the degradation product 3-hydroxybutyrate) and an increased activity of the immune system of the host.

In conclusion, our data demonstrated that 3-hydroxybutyrate is released from poly-β-hydroxybutyrate in sterile brine shrimp, and that this compound, at subinhibitory concentrations, inhibits several virulence-related phenotypes in pathogenic *V*. *campbellii*. Together with an induction of immune responses of the host, these data explain the protective effect of poly-β-hydroxybutyrate against infections.

## Methods

### Bacterial growth conditions

*V*. *campbellii* ATCC BAA-1116 was routineously grown in Marine Broth (Difco Laboratories, Detroit, USA) at 28 °C with shaking (100 rpm). Cell densities were determined spectrophotometrically at 600 nm.

### Axenic hatching of brine shrimp (*Artemia franciscana*)

All brine shrimp tests were performed with high quality hatching cysts of *Artemia franciscana* (EG^®^ Type, batch 6940, INVE Aquaculture, Baasrode, Belgium). Two hundred mg of cysts were hydrated in 18 ml of tap water during 1 h. Sterile cysts and nauplii were obtained via decapsulation, adapted from the protocol described previously^40^. Briefly, 660 µl of NaOH (32%) and 10 ml of NaOCl (50%) were added to the hydrated cyst suspension. The decapsulation was stopped after 2 min by adding 14 ml of Na_2_S_2_O_3_.7H_2_O (10 g l^−1^). During the reaction, 0.22 µm filtered aeration was provided. The decapsulated cysts were washed with autoclaved artificial seawater containing 35 g l^−1^ of Instant Ocean synthetic sea salt (Aquarium Systems Inc., Sarrebourg, France). The cysts were resuspended in a 50 ml tube containing 30 ml of autoclaved artificial seawater and hatched for 30 h on a rotor (4 min^−1^) at 28 °C with constant illumination (2000 lux).

### Determination of 3-hydroxybutyrate levels in poly-β-hydroxybutyrate-fed brine shrimp larvae

1000 mg l^−1^ stock solutions of poly-β-hydroxybutyrate were prepared by dissolving 500 mg sterile poly-β-hydroxybutyrate particles (98% poly-β-hydroxybutyrate, 2% poly-β-hydroxyvalerate; average diameter 25 µm; Goodfellow, Huntingdon, Ireland) into 500 ml of sterile artifical seawater. The suspensions were sonicated in a sonicater bath for 30 minutes. Axenic brine shrimp larvae were stocked in Schott bottles containing 500 ml sterile artificial sea water and mixed with the poly-β-hydroxybutyrate stock suspension to obtain different concentrations of poly-β-hydroxybutyrate particles. The bottles were provided with sterile (0.22 µm filtered) aeration and incubated for 24 h at 28 °C. Subsequently, the brine shrimp larvae were harvested on a 150 µm sieve and washed with distilled water. The bottom of the sieve was dried with paper cloth to remove excess water, and 350 mg samples of the brine shrimp larvae were transferred into eppendorf tubes, immediately frozen in liquid nitrogen and stored at −80 °C until further use. 3-hydroxybutyrate levels in the samples were determined with the β-Hydroxybutyrate (Ketone Body) Colorimetric Assay Kit (Cayman Chemicals, USA). Briefly, 1 ml of assay buffer (1 metros-HCl, pH 8,5) was added to the brine shrimp samples. The suspensions were mixed for 40 seconds using a vortex mixer (Ultrathurax), and subsequently centrifuged in a table-top centrifuge at 14500 rpm for 5 minutes. The supernatants were filtered over 0.22 µm filters and transferred into new eppendorf tubes. The 3-hydroxybutyrate concentration in the samples were determined as described in the manufacturer’s instructions. The 3-hydroxybutyrate concentration in the brine shrimp gut was calculated as follows. Assuming a dry weight of 5 µg per brine shrimp larva^41^, the samples contained approximately 7000 larvae. Further, Gunasekara *et al*. reported that the gut volume of a brine shrimp larva is approximately 0.002 µl^42^. Therefore, the total gut volume in the samples was 14 µl. The 3-hydroxybutyrate in the brine shrimp gut was then calculated assuming that all 3-hydroxybutyrate that was present in the 1 ml samples originated from the 14 µl total gut volume of the brine shrimp larvae used to prepare the samples.

### Determination of the impact of 3-hydroxybutyrate on the growth of *Vibrio campbellii*

*V*. *campbellii* was grown overnight in Marine Broth on a shaker at 28 °C. Subsequently, the strain was transferred 1:50 (v/v) to either undiluted Marine Broth or 50-fold diluted Marine Broth supplemented with salts to reach the same salt concentration as in undiluted Marine Broth (denoted nutrient-rich and nutrient-poor conditions, respectively). The media were supplemented with β-hydroxybutyrate at different concentrations (0, 1, 5, 25 and 125 mM). The pH of all solutions was adjusted to 6 or 7 by adding HCl. After inoculation, the suspensions were incubated at 28 °C in a static mode. Growth was monitored by measuring the optical density (OD_600_) during 24 h. Each treatment was performed in triplicate.

### Determination of the impact of 3-hydroxybutyrate on hemolytic, lipase, phospholipase and protease activities of *Vibrio campbellii*

Hemolysin, lipase, phospholipase and protease activities were determined as described previously^43^. Hemolytic activity was determined by supplementing Marine agar containing different concentrations of 3-hydroxybutyrate at pH 6 or 7 with 5% defibrinated sheep blood (Oxoid, Basingstoke, Hampshire, UK). Lipase activity was tested by adding 1% Tween 80 (Sigma-Aldrich) into marine agar containing different concentrations of 3-hydroxybutyrate at pH 6 or 7. Phospholipase activity was assessed on Marine agar supplemented with 1% egg yolk emulsion (Sigma – Aldrich), and different concentrations of 3-hydroxybutyrate at pH 6 or 7. Protease assay plates were prepared by mixing double strength Marine agar containing different concentrations of 3-hydroxybutyrate at pH 6 or 7 with 4% skim milk powder (Oxoid, Basingstoke, Hampshire, UK), autoclaved separately at 121 °C for 5 minutes to prevent denaturation of the protein. Clearing (hemolysin and protease) or opalescent (lipase and phospholipase) zones were measured after 3 days of incubation at 28 °C. The activity zones were divided by the respective colony diameters in order to correct for possible differences in growth. In those cases where there was no activity zone extending beyond the colony diameter, the activity zone was set equal to the colony diameter because in such case it is not possible to accurately determine the activity zone.

### Determination of the impact of 3-hydroxybutyrate on biofilm formation and exopolysaccharide production by *Vibrio campbellii*

Biofilm formation and exopolysaccharide production were determined as described previously^44^. A grown culture of *V*. *campbellii* in Marine broth at an OD_600_ of 1 was inoculated 1:10 (v/v) into Marine Broth containing different concentrations of 3-hydroxybutyrate at pH 6 or 7, and 200 µl aliquots of these suspensions were pipetted into the wells of a 96-well plate. Then, the bacteria were allowed to adhere and grow without agitation for 24 h at 28 °C. After that, the cultures were removed and the wells were washed three times with 300 μl sterile physiological saline (9 g l^−1^ NaCl) to remove all non-adherent bacteria. The remaining attached bacteria were fixed with 150 μl of 99% methanol per well for 20 min, after which the methanol was removed and plates were air-dried. Then, biofilms were stained for 15 min with 150 μl of a 1% crystal violet solution (Pro-lab Diagnostics, Richmond Hill, ON, Canada) per well. Excess stain was rinsed off by placing the plate under running tap water, and washing was continued until the washings were free of the stain. After the plates were air dried, the dye bound to the adherent cells was resolubilized with 150 μl of 95% ethanol per well, and absorbance was measured at 570 nm. Sterile medium served as negative control. For the quantification of exopolysaccharides, Calcofluor white staining (Sigma-Aldrich) was used. In brief, wells were rinsed after 24 h biofilm formation and 100 μl phosphate buffered saline containing 0.5 μl 5 mM Calcofluor white staining dye was added to the wells. After 60 min, fluorescence (excitation 405 nm and emission 500 nm) was measured with a Tecan Infinite M200 multi-reader.

### Determination of the impact of 3-hydroxybutyrate on swimming motility of *Vibrio campbellii*

Swimming motility assays were performed on soft agar (Marine Broth containing 0.2% agar) containing different concentrations of 3-hydroxybutyrate (0, 1, 5, 25 and 125 mM) and at pH6 or pH7, as described previously^36^. *V*. *campbellii* was grown overnight in Marine Broth, and 5 µl of the culture was spotted in the center of the soft agar plates. Plates were incubated for 24 hours, after which the diameters of the motility halos were measured. All assays were done in triplicate.

### Brine shrimp challenge test

Challenge tests were performed as described previously^45^. Briefly, after hatching, groups of 20 nauplii were transferred to new sterile 50 ml tubes that contained 20 ml of autoclaved artificial seawater with different concentrations of 3-hydroxybutyrate (0, 25 and 125 mM, respectively) (Sigma-Aldrich, Bornem, Belgium) and set at pH7. The animals were fed with *Aeromonas* sp. LVS3 at 10^7^ cells ml^−1^, and for the challenge treatments, tubes were inoculated with *V*. *campbellii* at 10^7^ cells ml^−1^. Subsequently, the falcon tubes were put back on the rotor and kept at 28 °C, and survival was scored after 2 days. All manipulations were done under a laminar flow hood in order to maintain sterility. Each treatment was done in triplicate.

### Quantification of brine shrimp-associated vibrios

Bacterial numbers associated with brine shrimp larvae were determined as described previously^45^. Briefly, rinsed larvae were homogenised with a pestle and sharp sand, followed by 1 min of bead beating. The homogenised samples were serially diluted and spread-plated on Marine agar.

### Statistics

Data analysis was carried out using the SPSS statistical software (version 15). Unless stated otherwise, all other data were compared with independent samples t-tests.
